# Placement of hemodialysis catheters with the help of the micropuncture technique in patients with central venous occlusion and limited access

**DOI:** 10.3906/sag-2006-11

**Published:** 2021-02-26

**Authors:** Erdem BİRGİ, Hasanali DURMAZ

**Affiliations:** 1 Department of Radiology, University of Health Sciences, Dışkapı Yıldırım Beyazıt Training and Research Hospital, Ankara Turkey

**Keywords:** Central venous catheterization, hemodialysis, jugular veins, occlusion

## Abstract

**Background/aim:**

This study aims to describe the technical success of the micropuncture technique, which is performed in placement of tunneled hemodialysis catheters in patients with central venous occlusion and limited access.

**Materials and methods:**

A total of 25 patients with central venous occlusion and in need of catheter placement for hemodialysis between 2012 and 2018 were included in this study and analyzed retrospectively. Technical success was defined as the placement of tunneled dialysis catheters with optimal position and function.

**Results:**

Internal jugular vein access in 16 patients (14 right and 2 left) and right subclavian vein access in 3 patients were successfully performed in placement of the tunneled dialysis catheter. Although internal jugular and subclavian vein access was attempted bilaterally, the procedure failed in 6 patients. The overall technical success of recanalization of the occluded central veins was 76% (19/25). No minor or major complications were encountered.

**Conclusion:**

Tunneled dialysis catheter placement through the occluded internal jugular and subclavian veins with the micropuncture technique is effective and safe in patients with limited vascular access. The recanalization of the occluded conventional access routes should always be kept in mind to allow for the preservation of vascular accesses for future requirements.

## 1. Introduction

The use of tunneled hemodialysis catheters, especially in patients with end-stage renal disease and with limited access for surgical fistulas, is increasing owing to its practical placement and management [1]. Central venous catheterization is one of the most common procedures performed in interventional radiology units because of its low complication rates, high technical success, and long-term patency [2,3]. A history of prior multiple catheter placements and long-term indwelling catheters can result in central venous stenosis or occlusion, and this can result in limited vascular access for dialysis sessions [3]. 

Internal jugular veins, subclavian veins, and femoral veins are accepted as the conventional access sites for tunneled catheters [2]. In case of occlusion or adversity while accessing the veins mentioned above, alternative or unconventional venous access routes, including translumbar inferior vena cava, transhepatic inferior vena cava, small venous collaterals, and recanalization of occluded central veins, can be used [4–8]. Also, the sharp puncture technique for recanalization of chronic central venous occlusions has been described for patients that could not be accessed with a guidewire [9]. 

Recanalization of the conventional veins in patients with central venous occlusion and limited access are primarily preferred owing to their superficial location and because it is easily accessible, whereas unconventional access routes such as translumbar inferior vena cava and hepatic veins are accepted as technically challenging and time consuming [2].

The purpose of this study is to describe the technical success of the micropuncture technique, performed in placement of tunneled hemodialysis catheters in patients with central venous occlusion and limited access.

## 2. Materials and methods

### 2.1. Patients

**Figure 1:**
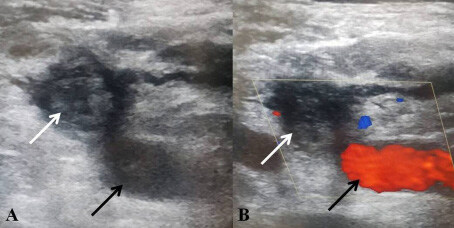
Subacute thrombus in IJV (white arrow). The adjacent patent CCA (black arrow) is detected during preprocedural planning with B-mode US (A) and color Doppler US (B). *IJV: Internal jugular vein; CCA: Common carotid artery.

A total of 25 patients with central venous occlusion and in need for catheter placement for hemodialysis between 2012 and 2018 were included in this study. Patients from hemodialysis centers were mainly referred to us to manage dysfunction of the current tunneled dialysis catheters or to place a new dialysis catheter in order to use it again. All patients had a history of end-stage kidney disease, multiple previous catheter placements, and a necessity for urgent dialysis. Low-molecular weight heparin was started in all patients before the procedure. The recanalization procedure was performed immediately due to the urgency of the need for dialysis. Doppler ultrasonography (US) was primarily used to detect the patency of the veins before planning the recanalization and catheter placement (Figures 1A and 1B). The age of the thrombus was determined with respect to the findings in Doppler US, whereas fluoroscopy during the procedure or contrast-enhanced computed tomography before the procedure were used in detection of thrombus length. Anechoic or minimal echogenic thrombus with venous distension was defined as acute, whereas echogenic thrombus with normal or minimal reduced calibration of vessel was defined as subacute thrombosis. Sonographic findings in the chronic stage of thrombosis are decreased vein diameter, diffuse thickening of the vein wall, and echogenic fibrous synechiae or bands [10]. Occlusions extending to the middle part of the brachiocephalic vein were classified as short and those extending to the vena cava superior were classified as long segment.

The main inclusion criteria for recanalization of occluded central veins were having no surgical upper limb dialysis options (e.g., upper limb arteriovenous fistula or graft) and limited access sites (occluded femoral veins, inferior vena cava, jugular and subclavian veins, history of multiple femoral catheter dysfunctions, and high infection risk due to femoral catheterization) for catheter placement. 

Patients with chronic central vein occlusions accompanying collateral vessels, uncorrected coagulation disorders, and severe orthopedic disorders preventing supine position were excluded from the study.

All patient data included in our study, such as demographic information and clinical findings, were obtained from medical records and from our procedure form. Informed consent forms were signed before treatment, and the ethics committee approved our study design.

### 2.2. Technique

**Figure 2:**
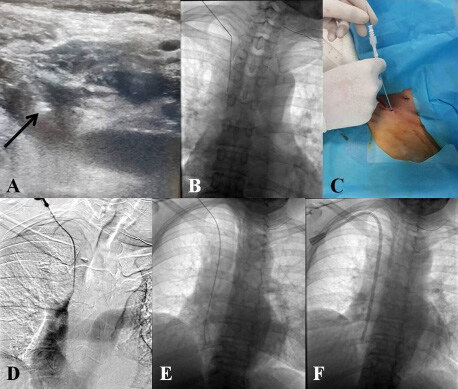
Endovascular recanalization procedure steps: Puncture of the occluded IJV under US guidance and echogenic tip of the micropuncture (21-G) needle (black arrow) (A); Delivering a 0.018-inch guidewire through the needle (B); Advancing 4-F sheath into the thrombosed IJV through the microguidewire (C); Patent lumen proved by injection of contrast medium (D); Advancing a 0.035-inch J–tip guidewire through the 4-F sheath (E); Positioning of the central venous catheter after gradual dilation and placement of the peel-away sheath (F). *IJV: Internal jugular vein; US: Ultrasonography; F: French.

Patients with coagulation parameters within normal limits (INR < 1.5, PLT > 50.000/mm3) were accepted for the procedure. All procedures were performed in the interventional radiology unit under the guidance of ultrasonography (Logiq S6, GE Healthcare, USA) and fluoroscopy (Artis Zee Floor DSA device, Siemens, Germany). Patients were put in supine position on the C-arm fluoroscopy table, and sterile conditions were provided. Neither sedation nor general anesthesia was used during the procedures; only local anesthetic agent (10-mL lidocaine 2%) was applied to the puncture region. Just above the clavicles, a 21-gauge (G) needle with an echogenic tip was inserted into the thrombosed segment of the internal jugular vein under US guidance, enabling the most parallel route to the longitudinal axis of the vessel as possible. In patients with failure of access via internal jugular veins bilaterally, a subclavian vein puncture was performed. A 0.018-inch × 45-cm nitinol guidewire was delivered through the needle under fluoroscopy, advanced into the patent central vein (vena cava superior) and right atrium after crossing the thrombosed segment. Alternatively, a 0.018-inch hydrophilic guidewire (ControlWire V18, Boston Scientific, Marlborough, MA, USA) was used in cases when there was difficulty in crossing long-segment occlusions. A 4-French (F) sheath (Coaxial Micro-Stick, Medcomp,  Harleysville, PA, USA) was advanced into the thrombosed internal jugular vein through the microguidewire. After withdrawing the guidewire, the distal tip of the sheath was confirmed as being in the patent lumen by injection of contrast medium. In order to provide extra strength and stability, a 0.035-inch J–tip guidewire (Amplatz Super Stiff, Boston Scientific) was delivered through the 4-F sheath and preferably advanced to the inferior vena cava in order to avoid serious cardiac arrhytmia. Gradual dilation was performed with the use of 8-F, 10-F, 12-F, and 14-F dilators before placement of the peel-away sheath of the dialysis catheter. In patients exhibiting difficulty in gradual dilation, a balloon with 5-mm diameter was used for the luminal patency needed for catheter placement. After maintaining vascular access with a peel-away sheath, the procedure was concluded in the same way as a routine tunneled dialysis catheter placement. At the end of procedure, the function and position of the catheters (Arrow Cannon II Plus Hemodialysis Catheter, 15F × 24/28/32 cm, Teleflex Medical, Wayne, PA, USA) were checked under fluoroscopy, and patients were discharged with recommendations for post-procedure catheter care. The recanalization and catheterization steps are demonstrated in Figures 2A–2F. No routine antibiotic prophylaxis was administered during the procedures.

Technical success was defined as the placement of the tunneled dialysis catheter in optimal position and function. We define the optimal position as being the location where the tip of the catheter placed within the junction of the vena cava superior and right atrium. Clinical success was defined as resumption of normal dialysis for at least 1 session. The CIRSE classification system was used in defining the complications [11]. 

### 2.3. Statistical analysis

Numeric data were presented as average, standard deviation, median, maximum, and minimum, while categorical data were pre­sented as number and percentage. Statistical data editing and analysis were per­formed using SPSS (Statistical Package for the Social Sciences v. 25.0, IBM Corporation, Armonk, NY, USA).

## 3. Results

A total of 25 patients [10 female (40%) and 15 male (60%); mean age: 54.84 years; range: 30-80 years; SD: 12.71 years] with central vein occlusion and a history of multiple dialysis catheter placements were retrospectively analyzed in this study. The age of the thrombus in the occluded segment demonstrated by Doppler US were acute in 3 patients (12%) and subacute in 22 patients (88%). The length of the thrombus in the occluded segment demonstrated by fluoroscopy during the procedure or contrast enhanced computed tomography before the procedure were short segment in 17 patients (68%) and long segment in 8 patients (32%). In our study, external jugular vein accesses were not feasible for cannulation due to insufficient vessel diameter or thrombosis. Considering femoral vein access: 8 patients (32%) had a nonfunctioning femoral catheter due to chronic femoral vein or vena cava inferior occlusion, 4 patients (16%) had an infection of femoral catheters, and 13 patients (52%) refused femoral catheterization due to difficulty of care and high risk of infection. In all patients, right internal jugular vein puncture was performed as the first choice due to its straight course to the right atrium. Internal jugular vein access in 16 patients (14 right and 2 left) and right subclavian vein access in 3 patients were successfully performed in placement of the tunneled dialysis catheter. Although internal jugular and subclavian vein accesses were attempted bilaterally, recanalization failed in 6 patients (24%). In 4 of the 6 patients, central venous catheters were placed via femoral vein despite high infection risk and 2 of the 6 patients were referred for surgical lower limb dialysis access. Overall, technical success in recanalization of the occluded central veins was 76% (19/25). Considering the age of occlusion, successful recanalization rates in acute and subacute thrombus were calculated as 100% (3/3) and 72.7% (16/22), respectively. The procedure was successful in 50% (4/8) of patients with long-segment occlusion and 88.2% (15/17) of patients with short-segment occlusion. During the procedures, no minor or major complications were encountered. Patients were followed up with respect to catheter dysfunctions and survival. Median follow-up time in 19 patients with successful recanalization and catheterization was 7.95 months (range: 5-12 months; SD: 1.95). During follow-up, catheter dysfunction and catheter-related infection were observed in 5 (26.3%) and 4 patients (21%), respectively. In these cases, catheters were exchanged through a guidewire from the same access point, and patients were discharged with functional central venous catheters. The primary patency rate was 78.9% at 6 months. Four patients died due to comorbid diseases during follow up. Patients’ demographic, central venous occlusion, and catheterization-related data are shown in the Table.

**Table:** Patients’ demographic, central venous occlusion, and catheterization-related data.

Patients		
Female	10	40%
Male	15	60%
Mean age (range)	54.84 years (30–80 years)	
Doppler US or fluoroscopy or enhanced computed tomography findings		
Acute thrombus	3	12%
Subacute thrombus	22	88%
Short segment	17	68%
Long segment	8	32%
Central venous access		
Right IJV	14	73.7%
Left IJV	2	10.5%
Right SCV	3	15.8%
Median follow up (range)	7.95 months (5–12 months)	
Patency rate at 6 months		78.9%

## 4. Discussion

Several methods, such as native arteriovenous fistula, arteriovenous grafts, and central venous catheters, have been defined for hemodialysis process [12]. Central venous tunneled catheter placement in patients with end-stage renal disease and whose surgery offered no opportunity to create an upper limb arteriovenous fistul/arteriovenous graft or presented the required time for fistula maturation is essential for continuous dialysis sessions [2]. Central venous catheterization is of critical importance not only for patients with end-stage renal disease but also for all chronic patients needing easy venous access for medication or nutrition [13]. Central venous stenosis, thrombosis, and infection are accepted as inevitable results of prolonged hemodialysis, especially in patients with comorbidities [14,15]. The role of interventional radiology has been expanding in parallel with the increasing need for dialysis worldwide, either in the placement of dialysis catheters and management of the catheter dysfunctions or for catheter-related infections. 

In the literature, several venous access routes have been defined in which to place a tunneled dialysis catheter. Although there is no consensus on which vascular access is best, clinical history, venous anatomy, and preference of clinician and operator are the decision-making parameters during planning [2]. In our interventional radiology unit, internal jugular vein access is preferred as the first choice to reduce several complications such as thrombosis and dysfunction. 

Beside the conventional venous access routes, salvage techniques, such as translumbar inferior vena cava access, transhepatic inferior vena cava access, catheterization of small venous collaterals, and recanalization of occluded veins, were reported [4]. As each access option has several advantages and disadvantages, operator experience is the main factor in determining the optimal salvage technique.

Recanalization of the occluded veins in catheter placement was first described in 1996 by Ferral et al. in 6 patients. In their study, a femoral vein approach was primarily used to get venous access. Before recanalization of the occluded innominate and subclavian veins, a 10-mm Amplatz Goose Neck snare was placed at the subclavian and supraclavicular area position as a target. Under fluoroscopy guidance, a 21-G needle was directed to the snare. Following the confirmed position of the needle, a 0.018-inch guidewire was advanced into the snare and through-and-through access was provided. With the support of through-and-through access, the central venous catheter was placed after dilation of the occluded vein [16]. In our study, no femoral venous access was required during the procedure in patients with successful recanalization of occluded internal jugular or subclavian veins. The micropuncture technique used in upper central veins is feasible even in patients with occluded femoral veins and vena cava inferior. 

In 2016, Too et al. reported about a technique called REBORN, which entailed the recanalization and balloon-oriented puncture for reinsertion of a dialysis catheter in nonpatent central veins. In their technique, a balloon is used as a target of the puncture to access the central vein. [17]. In their study, an angioplasty balloon is used as part of their technique; however, we needed angioplasty only in patients having difficulty in gradual dilation. 

The micropuncture technique for vascular access offers a small puncture site, less vascular injury, and the opportunity to initiate multiple punctures with the help of a 21- (G) needle when compared with the standard 18-G vascular access needle [18]. 

Besides the diameter of the micropuncture needle, the echogenic tip design also improves visualization under ultrasonography guidance, increasing the success rate and minimizing complications [19].

Other reasons for our 21-G needle preference are that the 0.018-inch guidewire in the micropuncture set allows it to be easily manipulated due to the short length of the guidewire, and it has better torque capability while crossing thrombosis.

Studies concerning the comparison of the micropuncture needle (21-G) and regular needles (18-G or 19-G) have been frequently performed during femoral arterial access [20,21]. Gutzeit et al. reported slightly a lower number of pseudoaneurysms in the micropuncture group; however, no significant decrease in complication rates was observed [20]. 

During central venous cannulation, inadvertent carotid artery puncture with standard needles might lead to a mortal hemorrhage in the neck region. Micropuncture needles reduce hemorrhagic complications, even in patients with uncorrected coagulopathy [18].

Retrograde recanalization of central venous occlusions via femoral vein route offers a lower risk of vascular complications, owing to the anatomic features of the femoral puncture site, as well offering a straighter path when compared with antegrade recanalization via internal jugular or subclavian veins [22]. Complications related with the canulation of upper central veins include: hemothorax, pneumothorax, carotid artery injury, mediastinal hematoma, and cardiac tamponade. As in our study, antegrade recanalization of upper central veins with the help of a micropuncture needle prevents such complications; thus, it can be used safely in patients with end-stage renal disease and limited vascular access for dialysis.

The major limitations of our study are retrospective design, the small number of patients, and lack of long-term patency rates and overall survival rates. The small number of patients included in the study is due to the fact that patients with central venous occlusion and limited vascular access are less commonly seen than patients undergoing standard central venous catheterization. 

In conclusion, tunneled dialysis catheter placement through the occluded internal jugular and subclavian veins with the micropuncture technique is effective and safe in patients with limited vascular access. We believe that maximum care and time should be spent for patients with end-stage renal disease due to limited vascular access sites for catheter placement. The recanalization of the occluded conventional access routes should always be kept in mind to allow the preservation of vascular access for future requirements.

## Funding

This research received no specific grant from any funding agency in the public, commercial, or not-for-profit sectors.
